# Systematic review practices in surgical disciplines

**DOI:** 10.1308/rcsann.2025.0003

**Published:** 2025-03-25

**Authors:** V Sahni

**Affiliations:** Research & Evidence (RF&E), New Delhi, India

Sir,

I write further to a paper published in *The Annals* entitled “Metastases from gastric cancer presenting as colorectal lesions: a report of two cases and systematic review”.^1^

It might have been a good idea to not label the said review as ‘systematic’ since it quite evidently does not comply with the PRISMA 2020 guidelines.^2^ The abstract does not provide a clear objective, eligibility criteria, methods used to determine the risk of bias or result synthesis. There is also no information regarding evidence limitations, funding or registration in the abstract.

The report itself lacks sections on introduction, methods and results.^1^ There is no defined objective or research question, no explicit eligibility criteria, the search strategy is inadequate, there is no information on data collection methods, and there is an absence of risk of bias determination and effect measures, amongst other factors which contribute to the rigour of a systematic review.

It is imperative to correctly identify the kind of review to be conducted and follow best practice in terms of execution as well. Studies either mislabelled or inadequately executed can potentially skew scientific literature and may lead to controversies.
